# Sex Steroids and Bone Health Status in Men

**DOI:** 10.1155/2012/208719

**Published:** 2012-10-24

**Authors:** Kok-Yong Chin, Soelaiman Ima-Nirwana

**Affiliations:** Department of Pharmacology, Faculty of Medicine, Universiti Kebangsaan Malaysia, Jalan Raja Muda Abdul Aziz, 50300 Kuala Lumpur, Malaysia

## Abstract

Male osteoporosis is a health problem which deserves more attention as nearly 30% of osteoporotic fractures happen in men aged 50 years and above. Although men do not experience an accelerated bone loss phase and testosterone deficiency is not a universal characteristic for aged men, osteoporosis due to age-related testosterone deficiency does have a negative impact on bone health status of men. Observations from epidemiological studies indicate that elderly men with higher testosterone can preserve their BMD better and thus are less prone to fracture. Observations on men with estrogen resistance or aromatase deficiency indicate that estrogen is equally important in the maintenance of bone health status. This had been validated in several epidemiological studies which found that the relationships between estrogen and bone health indices are significant and sometimes stronger than testosterone. Studies on the relationship between quantitative ultrasound and bone remodeling markers suggest that testosterone and estrogen may have differential effects on bone, but further evidence was needed. In conclusion, both testosterone and estrogen are important in the maintenance of bone health in men.

## 1. Introduction

Male osteoporosis is a health issue that deserves more attention. Although men do not experience a phase of accelerated bone loss similar to menopause of women, their bone health status declines gradually with age [1, 2]. The incidence of osteoporotic fracture in men increased exponentially after their seventies, which was relatively late compared to women in which the increase transpired as early as in their fifties [3, 4]. As a result of this late manifestation of osteoporosis and a relatively shorter life span compared to their female counterpart, it was generally thought that male osteoporosis was a rare disease condition [5]. However, the incidence of male osteoporosis proved otherwise. According to an estimation by Johnell and Kanis, in the year 2000, 39% of the global incidence of osteoporotic fractures happened in men. The incidence in men aged 50 years and above was 39% vertebral fractures, 30% hip fractures and 25% wrist fractures [6]. 

 Apart from that, male fracture patients seldom receive treatment for osteoporosis. The morbidity and mortality of male fracture patients are higher compared to female patients. The Dubbo Osteoporosis Study indicated that the 5-year mortality rate for male fracture patients aged 60 years and above was higher compared to females, and the mortality rate after a second fracture also exceeded that of females [7]. In the Canadian Multicenter Osteoporosis Study, it was reported that male fracture patients suffered from inability to take care of themselves and immobility [8]. Male osteoporosis also causes substantial economic burden to the health care system. According to estimation by Burge et al., men contributed to 24% of osteoporotic fractures in the United States, and it translated to an economic loss of 4.1 billion USD. The cost of osteoporosis will continue to rise as a result of increase in life span and in fracture incidence [9]. 

## 2. Testosterone and Bone Health Status in Men

Androgens are C-19 steroids produced by the testes and adrenal glands, while testosterone (T) is the most abundant androgen found in the body of men. Testosterone is bound by sex hormone-binding globulin (SHBG) and albumin in the blood. The bond between T and albumin is weak and dissociates readily when T reaches target cells. Hence, the amount of T bound to albumin and the truly unbound T (free fraction) is termed bioavailable T. The bond between T and SHBG is strong, thus preventing the entry of T into target cells. Testosterone can be converted into 5a-dihydrotestosterone (DHT) found in peripheral tissues. It can be catalyzed by the aromatase enzyme estradiol. Testosterone and DHT bind with androgen receptors while estradiol binds with estrogen receptors. Both of these receptors can be found in bone tissue [1, 10]. 

 In vitro studies demonstrated that androgen could increase the proliferation and decrease the apoptosis of osteoblast via regulation of protein kinase B [11]. It also played a vital role in the process of mineralization, which is the late differentiation stage of osteoblast [12, 13]. Androgen also prevented parathyroid-induced osteoclast formation [14] and decreased bone resorption activity of osteoclast via deactivation of lysosomal enzymes [15]. Interleukin-1 (IL-1) and parathyroid-induced prostaglandin E-2 production was also hindered by androgen [16]. It exhibited similar effects on IL-6 production in bone marrow stromal cells [17]. 

 The importance of androgen on bone health in men can be observed in orchidectomized and genetically modified (androgen receptor knockout/ARKO) rat model. A study showed that the femoral bones for both rat models were characterized by low bone mineral density (BMD) and low trabecular bone volume. The rats also had lower cross-sectional midfemoral shaft area, cortical area, cortical thickness, and periosteal perimeter compared to normal rats. This could contribute to low bone formation rate, mineralizing rate, and periosteal osteoblast number in these rats [18]. In a study by Yarrow et al., orchidectomized young male rats exhibited reduced trabecular volume, number, and width, increased osteoid surface, and trabecular separation one month after orchidectomy, and the condition was reversed by supraphysiological testosterone injection [19]. In ARKO mice, thinning of trabecular bone and lowering of mineralization rate happened in young mice [20]. This indicated that androgen acted through the androgen receptor to exhibit its anabolic effects on bone. These animal studies consolidate the theory that androgen and its receptors are important in the maintenance of the male skeletal system. 

Osteoporosis is also a common characteristic for hypogonadal men, regardless of their age and the type of hypogonadism. Testosterone was proven to increase the BMD of hypogonadal men, regardless of the type of hypogonadism [21, 22]. Suppression of bone resorption markers in patients receiving testosterone treatment was seen after a 6-month treatment period [21]. A study by Benito et al. indicated that apart from BMD, improvements in micromagnetic imaging indices such as bone volume fraction, trabecular thickness, topological erosion index, and surface-to-curve ratio were observed in hypogonadal males receiving T therapy [23]. The significance of androgen on bone health in men was also seen in prostate cancer patients who have undergone androgen deprivation therapy (ADT). A review by Diamond et al. reported that the BMD of patients receiving ADT showed greater reduction rate compared to normal males. For example, the BMD reduction rate at the femoral neck for patients receiving ADT was 1.8%–2.3% per year, which was higher compared to normal men at 0.7% per year [24]. Prostate cancer patients who received ADT and survived five years following treatment had higher bone fracture risk compared to patients who did not receive ADT [25]. Besides that, the prevalence rates of osteoporosis and osteopenia are also higher in prostate cancer patients who received ADT compared to normal men. In a study by Bruder et al., 53% of the ADT-receiving patients with a mean age of 77 years were osteoporotic [26]. Judging from these observations on T-deprived men, it was evident that T was important in maintaining the integrity of the male skeletal system.

## 3. Age-Related Testosterone Deficiency and Bone Health Status of Men

The decline of T in men is gradual, and subnormal T level is not a universal characteristic for aged men. There are aged men who are in their eighth decade but still possessing bioavailable T level within the normal reference range of young men [27]. Thus, terms such as partial androgen deficiency, late-onset hypogonadism, and testosterone deficiency syndrome have been used to describe hypogonadism that developed in aged men [28]. The Baltimore Longitudinal Aging Study found that the prevalence of hypogonadism among men aged 50 years and above was 12% for the age group 50–59 years, 19% for the age group 60–69 years, 28% for the age group 70–79 years, and 49% for the age group 80 years and above (reference range for T was based on total T value of men aged 21–45 years) [29]. In studies comparing middle-aged men to elderly men or young men to elderly men, a linear decline in T levels, especially free and bioavailable T, was a common finding regardless of study types (cross-sectional or longitudinal) (refer to Table 1) [30–32].

 The age-related decline in testosterone level was attributed to two factors, which were the degeneration of Leydig's cells and the increase of SHBG level with age. In rat models, age increment was related to the decline of weight of the testes, volume of Leydig's cells, and T production by Leydig's cells [45]. Leydig's cells in aged rats were found to be less responsive towards luteinizing hormone (LH) stimulation and possess less hormone receptors compared to control rats [46]. The binding of LH to its receptor also produced relatively less cAMP in old rats compared to young rats [46]. A genetic study revealed that expression of genes responsible for cholesterol metabolism (Scavenger Receptor class B member 1 (SR-B1) and carboxylesterase ES-10), steroidogenesis (cytochrome P450scc and cytochrome P450c17), and antioxidant enzymes (copper-zinc superoxide dismutase and glutathione transferase) was reduced in aged rats compared to young rats [47]. From these results, it is reasonable to envisage that Leydig's cell degeneration is the result of a combination of various factors such as failure of the oxidative stress barrier, which leads to suppression of cAMP generation and damage to T producing enzymes. This causes a decreased response towards LH stimulation and subsequently the lowering of testosterone production. 

 Epidemiological observations showed that age-related decline of free and bioavailable T was greater than total T [30]. This is direct result of the increase of SHBG level with age. In normal young men, a rise in SHBG is usually followed by a feedback mechanism, in which the body would increase the T production to maintain the optimal level of free T. However, this feedback mechanism fails in aged men because their Leydig's cells are unable to synthesize sufficient T. As a result, the level of unbound T drops gradually [1, 27]. The increase of SHBG had been linked to age-related decline of growth hormone and insulin-like growth factor-1, as a negative association was found between these factors in cross-sectional studies [48, 49]. However, a direct link has not been validated. 

 The age-related decline in testosterone levels poses negative implications on bone health status of aged men. A study on American men aged 20–90 years by Paller et al. found that those with free T level at the lowest quartile had the highest probability to be osteopenic [42]. A study by Van Den Beld et al. also found that there was a positive and significant association between T levels (total, free, and bioavailable fraction and androgen index) and BMD in a population of aged men [34]. This suggested that aged men with higher T levels were better able to maintain their BMD. The Swedish Osteoporotic Fractures in Men (MrOS) Study discovered that there was a negative relationship between free testosterone and previous fracture history after the fifth decade of life in a population of aged men [38]. These findings were also found in non-Western populations. The Hong Kong MrOS study found a positive and significant relationship between femoral and vertebral BMD and free T in a population of Chinese men aged 65 years and above. The researchers also suggested that free T level at the lowest quartile was predictive of bone fracture [44]. This could be due to an increased incidence of fall and a decrease in body functional capacity related to T level [36]. A study by Khosla et al. found that bioavailable T was associated with the subendocortical area in men, which was an index of endocortical resorption. The relationship was strongest at the femoral neck and in the aged population compared to the young population [37]. However, discrepancies existed among different studies, in which some found no relationship between bone health status of men and total T level [42, 44]. There were even some studies which stated that all T levels were not related to male BMD [39]. Besides, the relationship between T and BMD was not consistent at different testing sites. For example, Venkat et al. found that T was related to BMD at the vertebrae but not at the femora [43]. 

## 4. Estrogen and the Male Skeletal System

The estrogen level in men was shown to be higher than in postmenopausal women [33, 50]. The estrogen hormone in men is produced via conversion of T to estrogen via the aromatase enzyme (cytochrome 19) [1]. About 15% of the estrogen in men originates from the testes while the other 85% comes from peripheral tissue inclusive of bone. Furthermore, aromatase enzyme was found in osteoblasts, osteocytes, chondrocytes, and adipocytes but not in osteoclasts [51]. Therefore, it is reasonable to postulate that estrogen produced in the bone of men has paracrine or intracrine function. Estrogen had been found to reduce apoptosis, oxidative stress, and nuclear factor kappa B (NF-*κ*B) activity in osteoblasts and increase apoptosis and hinder differentiation of osteoclasts [52]. 

 The traditional notion that estrogen is only important in maintenance of the female skeletal system while testosterone is vital for the male skeletal system is now challenged by several experiments of nature. Smith et al. reported a young man with a rare condition of estrogen resistance due to mutation in the gene coding for the estrogen receptor. The patient also had low BMD and incomplete closure of the epiphyseal plate [53]. Later, Morishima et al. reported a young osteoporotic man with elevated bone remodeling makers who suffered from aromatase deficiency due to a mutation in the genes coded for CYP19 [54]. Estrogen replacement was reported to successfully increase the BMD and T-score of patients with aromatase deficiency and to normalize their bone remodeling marker levels, which subsequently led to closure of the epiphyseal plate and increase in bone age [55–58]. In a report by Bouillon et al., the BMD increment in a young man with aromatase enzyme deficiency after receiving estrogen replacement therapy was due to increase in bone size, without any significant changes in volumetric BMD [59]. This shows that estrogen is as important as androgen in inducing periosteal apposition. 

 Animal studies also proved that estrogen was important in the maintenance of bone health. A study in male mice with deletion of the aromatase gene showed that after maturation, the male mice had a lower femoral bone mass compared to wild type mice. This was attributed to increased bone resorption in the trabecular bone of the genetically modified mice. In aged animals, significant reductions in cortical and trabecular bone mass were observed in genetically modified mice compared to wild type [60]. Other researchers also showed that deletion of the aromatase gene in mice resulted in higher endosteal bone resorption, higher osteoclast number, and lower trabecular bone volume [61]. 

 The age trend for estrogen in men is inconclusive. Several epidemiological studies found a decreasing trend with age [33, 34] while other studies found no significant changes [62] or a significant elevation with age [63]. Regardless of this, estrogen was found to be associated significantly with bone health status of elderly men in several large epidemiological studies. The Framingham Study discovered that aged men with higher estradiol level had higher BMD, and the difference in BMD between the first quartile and the fourth quartile was equivalent to 10 years of aging on bone [35]. Positive and significant relationships between estradiol level and several hip strength parameters, especially cross-sectional area of bone, were also observed in the Boston Bone Health Study [41]. Using QCT techniques, Khosla et al. discovered that E was significantly correlated with volumetric BMD and structural parameters, especially at cortical sites [37]. The American MrOS study also discovered that aged men with estradiol levels at the lowest quartile had significantly higher risk for nonvertebral fracture [40]. According to The Third National Health and Nutrition Survey of the United States, estradiol levels were found to be associated with BMD in older male population but not in the young population [42]. Hence, from these observations, estrogen seems to prevent deterioration of bone health status in elderly men but not in young men, particularly at the cortical sites. The findings of these epidemiological studies are summarized in Table 1.

## 5. Differential Effects of Testosterone and Estrogen

Bone health status can also be examined using methods other than BMD and QCT measurements. In a study using calcaneal quantitative ultrasound parameters as determinants of bone health status, both bioavailable T and bioavailable E were found to be associated with bone health status of men, but the effects of association of bioavailable E were stronger than bioavailable T. In the same study, bioavailable E had significant relationship with broadband attenuation of sound (BUA) but not with speed of sound (SOS) [64]. Another study by Chin et al. on Malaysian men found that the relationships between T measurements and SOS were significant but the relationships between E measurements and SOS were not [65]. Since BUA and SOS reflected different physical properties of bone [66], this might imply T and E influenced different bone properties. However, this hypothesis is yet to be validated. 

In a remarkable experimental study by Falahati-Nini et al., relative contributions of T and E in regulating bone remodeling in elderly men were assessed. It was found that E significantly contributed to suppression of bone resorption, but the contribution of T was insignificant. On the other hand, both T and E were found to have significant contribution towards bone formation [67]. This was in line with the cross-sectional observation of Fatayerji and Eastell in which T and E were significantly correlated with bone formation markers in a group of men aged 20–79 years [68]. 

## 6. Conclusion

Both E and T are indispensable for the maintenance of bone health status in men. The significance of these sex hormones on bone health was clearly depicted in male patients who suffered from deficiency of either hormones due to hormone deprivation therapy or genetic mutation. These observations challenge the traditional view that testosterone is the sole determinant of bone health status in men. It is still unclear whether they act synergistically or independently in the maintenance of skeletal integrity in men. More studies should be conducted to establish the role of T and E in regulating bone health in men. 

## Figures and Tables

**Table 1:** Epidemiological studies on the relationship between sex hormones and bone health status in men.

Researcher	Study and subject involved	Findings
Khosla et al. (1998) [33]	Rochester Epidemiology Study. 280 men aged 25–85 years.	Testosterone and estrogen (estradiol and estrone) correlated significantly with BMD of subjects at multiple sites. In a multiple stepwise regression model, estrogen was the only significant predictor of proximal femoral BMD.
van den Beld et al. (2000) [34]	403 men aged 73–94 years.	Testosterone and estradiol (total, free, and bioavailable fractions) were significantly associated with total body, femoral neck, ward and trochanteric BMD.
Amin et al. (2000) [35]	Framingham Study. 405 men aged 68–96 years.	Total estradiol had a positive and significant relationship with BMD of subjects while the relationship between testosterone and BMD was not significant.
Szulc et al. (2003) [36]	MINOS Study. 792 men consisting of two groups. Group 1 aged 19–40 years and Group 2 aged 51–85 years.	The reduction of free testosterone and free testosterone index showed a significant relationship with inability to perform functional tests and risk of fall. Men with lower BMD also had lower free testosterone.
Khosla et al. (2005) [37]	Rochester Epidemiology Study. 314 men aged 22–91 years.	Sex hormones (bioavailable testosterone and estradiol) had significant relationship with several volumetric BMD and structural indices as assessed using pQCT. Subendocortical area showed significant and negative relationship with testosterone. This indicated that testosterone exhibited antiresorptive effect in subendocortical area. Estrogen exhibited significant relationship with vBMD and structural indices particularly at cortical sites.
Mellström et al. (2006) [38]	MrOS Study Sweden. 2908 men with a mean age of 75.4 years.	Free testosterone level below the overall median was predictive of fracture after 50 years of age while free estradiol level at the lowest 10th percentile could predict fracture. Free testosterone and estradiol had significant relationship with BMD of subjects at multiple sites.
Araujo et al. (2008) [39]	Boston Area Health Study. 832 men aged 30–79 years.	Only estradiol levels correlated significantly with femoral neck, hip, ultradistal radius, and spine BMD after adjustment for confounders was performed. Testosterone did not correlate with BMD significantly.
LeBlanc et al. (2009) [40]	MrOS Study USA. 1436 Caucasian and 446 minorities aged 65 years and above.	Men with low bioavailable estradiol, low bioavailable testosterone, and high SHBG had significantly higher risk for nonvertebral fracture.
Travison et al. (2009) [41]	Boston Area Health Study. 808 men aged 30–79 years.	Estradiol levels had significant relationship with several hip strength parameters, especially cross-sectional area. It was suggested that anthropometric factors were the mediating factor in this relationship.
Paller et al. (2009) [42]	The Third National Health and Nutrition Survey USA. 623 men aged 20-90 years.	Men with the lowest quartile of free testosterone had 4-time odds of suffering from osteopenia. Subjects with the lowest quartile of free estradiol had 70% odds of suffering from osteopenia. The relationship between testosterone and bone health status was more obvious in aged men, but the relationship between estradiol and bone health status was apparent in younger men.
Venkat et al. (2009) [43]	350 Indian male military recruits aged 21–55 years.	Spine BMD had significant relationships with estradiol and testosterone (total, free, and bioavailable fraction) although the strength of the relationship was lower for testosterone. Femoral BMD was associated with estradiol but not testosterone levels.
Woo et al. (2011) [44]	MrOS Hong Kong. 1158 Chinese men aged 65 years and above.	Free testosterone level correlated significantly with femoral and hip BMD, but the percentage of changes of BMD in 4 years was significantly associated with estradiol, not testosterone. Men in the lowest quartile for bioavailable and free testosterone and estrogen were more prone to fracture.
